# Health-Related Telemonitoring Parameters/Signals of Older Adults: An Umbrella Review

**DOI:** 10.3390/s23020796

**Published:** 2023-01-10

**Authors:** José Félix, Juliana Moreira, Rubim Santos, Elina Kontio, Ana Rita Pinheiro, Andreia S. P. Sousa

**Affiliations:** 1 Department of Physics, School of Health, Polytechnic Institute of Porto, Rua Dr. António Bernardino de Almeida, 400, 4200-072 Porto, Portugal; 2 Center for Rehabilitation Research (CIR), School of Health, Polytechnic Institute of Porto, Rua Dr. António Bernardino de Almeida, 400, 4200-072 Porto, Portugal; 3 Department of Medical Sciences, University of Aveiro, Agras do Crasto, Campus Universitário de Santiago, 3810-193 Aveiro, Portugal; 4 Department of Physiotherapy, School of Health, Polytechnic Institute of Porto, Rua Dr. António Bernardino de Almeida, 400, 4200-072 Porto, Portugal; 5 Faculty of Engineering and Business, Turku University of Applied Sciences, Joukahaisenkatu 3, 20520 Turku, Finland; 6 School of Health Sciences (ESSUA), University of Aveiro, Agras do Crasto, Campus Universitário de Santiago, 3810-193 Aveiro, Portugal; 7 Institute of Biomedicine (iBiMED), University of Aveiro, Agras do Crasto, Campus Universitário de Santiago, 3810-193 Aveiro, Portugal

**Keywords:** elderly, biological signals, environmental signals, health telemonitoring, wearable sensors/devices, home base station

## Abstract

Aging is one of the greatest challenges in modern society. The development of wearable solutions for telemonitoring biological signals has been viewed as a strategy to enhance older adults’ healthcare sustainability. This study aims to review the biological signals remotely monitored by technologies in older adults. PubMed, the Cochrane Database of Systematic Reviews, the Web of Science, and the Joanna Briggs Institute Database of Systematic Reviews and Implementation Reports were systematically searched in December 2021. Only systematic reviews and meta-analyses of remote health-related biological and environmental monitoring signals in older adults were considered, with publication dates between 2016 and 2022, written in English, Portuguese, or Spanish. Studies referring to conference proceedings or articles with abstract access only were excluded. The data were extracted independently by two reviewers, using a predefined table form, consulting a third reviewer in case of doubts or concerns. Eighteen studies were included, fourteen systematic reviews and four meta-analyses. Nine of the reviews included older adults from the community, whereas the others also included institutionalized participants. Heart and respiratory rate, physical activity, electrocardiography, body temperature, blood pressure, glucose, and heart rate were the most frequently measured biological variables, with physical activity and heart rate foremost. These were obtained through wearables, with the waist, wrist, and ankle being the most mentioned body regions for the device’s placement. Six of the reviews presented the psychometric properties of the systems, most of which were valid and accurate. In relation to environmental signals, only two articles presented data on this topic. Luminosity, temperature, and movement were the most mentioned variables. The need for large-scale long-term health-related telemonitoring implementation of studies with larger sample sizes was pointed out by several reviews in order to define the feasibility levels of wearable devices.

## 1. Introduction

Digital technologies, such as smart wearable healthcare devices, are increasingly being used to support wellbeing, to encourage the independence of older adults, and to monitor health [1]. Telemonitoring, defined as the use of technologies for patient monitoring geographically separate from the health professional, at home, in healthcare units, and/or in hospitals [2], is currently viewed as a promising solution for older adults’ healthcare. The data obtained by this kind of system not only inform caregivers and healthcare professionals about abnormal changes, helping therefore in the early detection and management of a health condition, but they can also be used in the self-management of older adults, promoting appropriate changes to their daily routines or behavior [3].

Several technologies, operating under different technical specifications and algorithms, have been developed in recent years, with different properties and levels of validity and reliability [4]. Depending on each system, devices can be placed in different body regions, for example, the wrist, the chest, the fingers, and the ankle, allowing the measurement and monitoring of several biological signals, such BT, HR, RR, BP, StO_2_, and BG [5]. Considering the role of the environment in biological signals as well as on older adults’ health, the combination of biological signals’ monitoring together with environment monitoring would better characterize health conditions or even the risk for older adults. It is known that environmental conditions have a significant impact on older adults, such as the house design, the sources of temperature and the temperature itself, gas density, air saturation, and luminosity. In this sense, the development of solutions as central stations that allow the daily analysis of the environment has also increased in recent years [6,7,8,9].

There have been several systematic reviews of health-related biological and environmental signals, measured in different age groups of healthy people or those with pathological conditions. These reviews identified vital signals such as HR, BP, BT, gas density, and humidity but have not always identified their normative values, information about the participants’ related health status, the equipment and measurement method, or the validity or reliability values [10,11,12,13]. Technological advances have enabled the monitoring of several health-related biological signals in older adults, ranging from cardiovascular to movement-related signals. The specifications of the systems used vary in terms of size, portability, and normative values’ characterization, dependent on health and environment conditions. Therefore, it is important to systematically gather information about the health-related biological signals measured in older adults, their measurement method, equipment and psychometric properties, the normative values of the health-related biological signals, as well as information about the health status of the elderly. These data are useful for the decision-making process, based on the biological signals, the significance of health-related parameters extracted, the system usability, and the psychometric properties.

Hence, an umbrella review of the type and usage of telemonitoring technologies in older adults is needed. Accordingly, this study developed an umbrella review to gather the health-related biological and environmental signals and instruments from the most recent telemonitoring technologies used in older adults.

Abbreviations part contains a table with all the notations in this article.

## 2. Materials and Methods

This umbrella review was conducted in accordance with the guidelines of PRISMA and the guidelines for developing and summarizing umbrella reviews [14,15]. No ethical approval was needed as we used data from published studies. This umbrella review was registered in PROSPERO under the number CRD42021282273. The search was carried out in December 2021, and the screening process occurred between January and May 2022.

### 2.1. Eligibility Criteria

Systematic reviews and meta-analyses, published between 2016 and 2022, written in English, Portuguese, or Spanish, aiming to review telemonitoring health-related biological and environmental signals in older adults, were included. Studies referring to conference proceedings, ineligible articles, or articles with abstract access only were excluded.

### 2.2. Search Strategy

A protocol with the search strings for each scientific database, namely PubMed, the Cochrane Database of Systematic Reviews, the Web of Science, and the Joanna Brigs Institute Database of Systematic Reviews and Implementation Reports, was properly designed by the researchers prior to the search. The search included MeSH terms. The search string for each database is shown in the supplemental data, Appendix A. References of the systematic reviews were also analyzed to identify further possibly relevant articles. 

### 2.3. Data Collection and Analysis

#### 2.3.1. Selection Process and Data Extraction

The articles’ selection process involved two sequential phases, in which studies were independently reviewed by two reviewers (JF and JM). In case of doubt, another independent reviewer (ASP) was consulted; we excluded all studies that did not fit the criteria. In phase one, the selection was based on the analysis of the title and the abstract. In phase two, a full-text analysis was conducted.

Next, the data were extracted independently by two reviewers (JF and JM), using a predefined table form, consulting a third reviewer in case of doubts or concerns (ASP). The information extracted was organized into two domains. The first included the city and country of the review, as well as, when available, the city and country of the original studies; the number of the included studies; the number of participants included and their mean/range age; and the population (healthy or pathological condition) and the population context (community-dwelling/controlled lab/institutionalized). The second domain included information regarding the monitoring technology including the wearable type, sensor type, wearable/sensor location; the health-related biological signals; the health-related environmental signals; the psychometric properties of the outcome measures by body regions (validity and reliability); the cutoff of the biological and environmental signals; and the health status information of the measures of the biological signals and the environmental signals and the usability information. 

#### 2.3.2. Methodological Quality Assessment

The methodological quality assessment of the included reviews was performed independently by two reviewers (JF and JM). In case of disagreement, a third reviewer was consulted (ASP).

AMSTAR 2.0 was used [16]. This tool contains 16 items, which can be answered with “yes”, “partially yes”, and “no”. Depending on the score and how many critical and non-critical flaws an article had, it could be classified as “high quality”, “moderate quality”, “low quality”, and “critically low quality”. The critical domains of AMSTAR 2.0 are: “protocol registered before commencement of the review”; “adequacy of the literature search”; “justification for excluding individual studies”; “risk of bias from individual studies being included in the review”; “appropriateness of meta-analytical methods”; “consideration of risk of bias when interpreting the results of the review”; and “assessment of presence and likely impact of publication bias” [16].

## 3. Results

**Figure 1:**
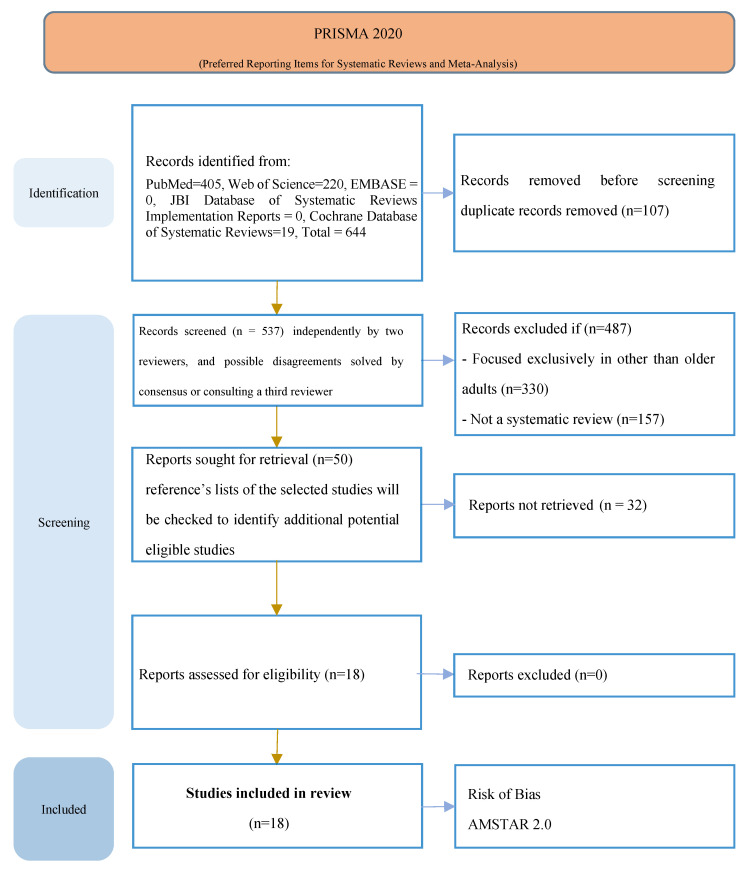
PRISMA Flowchart.

The database search retrieved 644 records, seven of them were duplicates, which were eliminated. After the analysis of titles and abstracts, 487 studies were excluded because they were not systematic reviews (n = 157) or they exclusively analyzed groups other than older adults (n = 330). Accordingly, 18 systematic reviews were included in this umbrella review (Figure 1). Nine of the reviews were developed in European countries [17,18,19,20,21,22,23,24,25], four in North American countries [26,27,28,29], three in Australia [30,31,32], one in Pakistan [33], and one in India [34]. The number of studies included in each review varied between 7 and 73, with an average of 32 studies, and a median of 25 studies. A detailed description of the studies is shown in Table 1.

### 3.1. Searched Databases by the Studies

The databases most commonly searched by the studies included MEDLINE (ten studies [17,19,21,22,23,26,28,30,31,33]), PubMed by nine [18,20,24,25,27,29,31,32,33]), EMBASE by eight [17,20,21,22,26,28,29,32], CINAHL by six [17,20,23,29,30,31], SCOPUS by five [19,23,24,25,34], COCHRANE by four [18,26,30,33], Web of Science by three [19,22,25], Science Direct [19,23], Google Scholar [29,33], ACM digital [19,24], IEE Xplorer [19,24], and Ovid by two [22,28], and APA PsycINFO [20], EBSCOhost [32], Academic Search Elite [19], Sport Discus [29], and AMED [30] by one. A survey report by the WHO on telecommunication and information technology was also referred to by one study [34]. A characterization of each study is shown in Table 1.

### 3.2. General Characterization of the Studies: Country Origin, Included Types of Study, and Their Methodological Quality

Some reviews indicated the countries of the included studies, whereas others indicated only the continent; seven reviews did not report the origin of the included articles (Table 1). 

Different types of studies were included in each review, varying between observational [19,21,22,23,24,26,28,30,32], CC [19], CSS [18,19,25], CO [19], RCT [17,21,22,28,32,33], nRCT [17], CS [28], PRO [27], and the development of a monitoring system [18].

The methodological quality of each systematic review is shown in Table 2. Only four reviews were considered high quality [21,22,29,32], five presented moderate quality [17,23,25,26,30], eight presented low quality [18,19,20,24,27,28,31,33], and one showed critically low quality [34].

### 3.3. Characteristics of the Studies’ Participants

The reviews included samples ranging from 290 to 17131 participants [17,18,19,20,21,22,23,24,25,26,27,28,29,30,31,32,33,34].

Nine of the reviews included older adults from the community as a sample, and the other studies also included older adults who were institutionalized in healthcare units and in a controlled environment. The participants were over 16 years old, and the oldest reported participant was 94. The reviews included studies involving healthy participants [19,20,22,23,24,26,29] and participants with different pathologic conditions, such as cardiovascular [19,20,21,24,26,27,28,30,31], neurologic [17,19,20,24,25,27,28], metabolic [19,20,22,24,26,27,28], respiratory [19,28,30,31], and other conditions [19,28,29,33]. A more detailed description of each study’s participants is shown in Table 3.

### 3.4. Health-Related Biological Signals and Body Regions

The most measured health-related biological signal was PA (steps, daily activity time, calories, PA level, energy expenditure, and movement), presented in 13 reviews [17,18,19,20,23,25,27,29,30,31,32,33,34], followed by other biological signals, namely HR [19,21,26,27,28,31,34], RR [19,28,31], ECG [19,28,31], BP [19,21,34], glucose levels [22,28], sleep [19,29,34], and fall risk [20,27].

The health information status and cutoffs for steps [17,19,20,23,27,29,30,31,34], PA level [17,18,19,20,23,30,31,33], movement [18,23,25,27,32], cardiac rhythm [21,26,31], and fall risk [20,27] were identified, while for energy expenditure [23,25,29,30,31], RR [19,28,31], and HR [19,21,26,27,28,31,34], the cutoff values could be extracted. A detailed characterization of the health-related biological signals is shown in Table 4.

The systems were placed in several body locations, including the wrist [20,23,25,26,29,30,31,33], waist [17,18,20,23,25,26,29,30,31], ankle [17,18,20,29,30,31,33], chest [17,25,26,28,31,33], arm [18,21,28], hip [23,29,33], thigh [17,25], pocket, bra, elbow, neck, and torso [20,23,29]. 

The number of steps was the variable assessed on more body locations (lumbar spine, upper arm, bra, torso, chest, sternum, tight, wrist, waist, and ankle), while the fall risk was assessed only on the torso, the BP on the arm, and the ECG on the chest.

It is important to note that a movement category considered all free-living activities. 

### 3.5. Sensor Types for Biological Signals Measurement

As described in Table 5, the systems used to monitor health-related biological signals in older adults included accelerometers most frequently [17,18,23,25,27,32,33,34], then ECG sensors [24,26,27,30], photoplethysmography sensors [26,34], BP devices [19,21,24,26,34], and others. The data transmission occurred through Bluetooth, wireless, and/or by using cables [17,18,19,20,21,22,23,24,25,26,27,28,29,30,31,32,33,34].

### 3.6. Psychometric Properties 

Only seven reviews indicated the psychometric properties of the systems, as shown in Table 6 [17,18,26,29,30,32,33]. Among the reviews, only steps, daily activity time, PA level, posture, sleep, energy expenditure, cardiac rhythm, and movement in free-living activities were assessed. Some of the presented variables were measured in different places, although their psychometric properties were only available for some body regions. Because different types of studies were included in different reviews, comparing the results was difficult [17,18,26,29,30,32,33].

The criterion validity was established for: steps (ranging from r = 0.76 (ankle) to r = 0.96 (wrist)) [17,18,31]; PA (ranging from r = 0.78 (waist) to r = 0.978 (hip)); posture (error = 40.31% (thigh)) [17,33]; sleep (error = 8.6% (wrist)) [17,33]; and energy expenditure (r = 0.74 (wrist), error < 8.6% (torso)) [29,30].

The reliability was established for: steps (ranging between an ICC of 0.15 (wrist) and 0.99 (ankle) [17,18,31], movement (ICC = 0.74 (hip) and ICC = 0.95 (waist)) [32], and cardiac rhythm accuracy ranging from 94% to 97% and specificity ranging from 87% to 100% for fingertips [26].

### 3.7. Environmental Signals

Only two reviews [24,34] reported unobtrusive in-home monitoring, allowing participants’ quality of life to be assessed using environmental signals and a home base station.

Passive infrared motion, contact, pressure and electrical current sensors were the most frequently used to monitor the participants’ behavior, measuring the presence in specific places or furniture or the time spent on activities [24].

Older adults’ health-related biological signals were measured through equipment placed in the environment, such as temperature, ECG and HR; however, other signals were also identified such as presence, activity, gas concentration, and sound. A more detailed description of the results of the environmental signals is presented in Table 7.

#### 3.7.1. Sensor Types Used to Measure the Environmental Signals

Several different types of sensors were identified in the review. The sensors included a contact sensor, a motion sensor, an electrical current sensor, a thermometer, a flowmeter, a camera, an infrared camera, a pressure sensor, a humidity sensor, a gas sensor (air quality and smoke), and other sensors, measuring the presence at home, the activity of daily living, the time on activity, the activity level, and several environmental data (temperature, humidity, gas, light, rain, and flame) [24,34]. A more detailed description of the results of the environmental signals is presented in Table 7.

#### 3.7.2. Location of the Measurement of the Environmental Signals

In the analyzed studies, different locations were identified for the placement of the sensors. The locations ranged from household appliances, such as kitchen equipment, to audiovisual equipment. Infrastructures such as walls and floors or even furniture were also mentioned. Finally, divisions in general were also mentioned, such as the kitchen, living room, or bedroom [24,34]. A more detailed description of the results of the environmental signals are present in Table 7.

#### 3.7.3. Psychometric Properties of Sensors Used to Measure the Environmental Signals

The psychometric properties were not reported.

## 4. Discussion

Aging and increased longevity are two of the greatest developmental difficulties in modern society. In the next 40 years, in Europe, it is projected that people over 65 years will be the fastest growing age group, leading to a doubling of the older adult population compared to the younger population [35,36]. This growth will imply an increase in care to maintain the quality of life of this population, considering the three strongest aspects of aging, namely the loss of autonomy, the increase in loneliness, and the management of acute or chronic health conditions [37]. Altogether, this represents an increase in total cost expenditures, as well as an intensification of healthcare or social care. The development of efficient methods and strategies to collaborate in the monitoring of the older adult population has been stated as essential to reduce accidents and traumatic events, manage chronic conditions, and increase older adults’ control over their health and quality of life, thus meeting the third objective of the 2030 agenda developed by the United Nations, which aims for good health and wellbeing [38,39,40]. This challenge motivated the development of the present review to understand the progress in monitoring older adults, namely which health-related biological and environmental signals are being used, as well as which instruments are being used to access them. 

Most of the reviews were performed in developed countries. This is in line with what is known in the scientific community; developed countries conduct more research for the maturation and development of scientific knowledge and are at the forefront of technological innovations and their applications [41]. However, it is relevant to note that two reviews were conducted in developing countries, and those countries have a strong presence in the release of scientific material to the international community [24,34]. The same trend was observed in the countries of the original studies included in each of the reviews, where European countries and North America were the most reported. Again, in these developed countries, the economic factor and the gross domestic product available for research are important.

All reviews indicated the databases searched varied between MEDLINE, PubMed, and EMBASE. These were the most inclusive databases that could assist in finding all available articles [42]. However, some recent articles have shown that it is advisable to conduct a review at least in EMBASE, MEDLINE, Web of Science, and Google Scholar to be inclusive, a fact that was not always fulfilled by the included reviews [42].

As would be expected, the health-related biological signals that appeared to be the most frequently measured in older adults corresponded to the vital signals [43,44,45]. However, other signals related to movement variables were frequently considered, the steps being most the frequent, followed by energy expenditure. Body temperature, peripheral oxygen saturation, fall risk detection, glucose levels, and weight were also assessed. The signals monitored were used to assess the daily activity time, PA level, posture, sleep, stress, energy expenditure, fall risk, and movement quality. Naturally, movement is one of the most studied biological signals due to its ease of acquisition through a wide range of accelerometers and movement sensors, thus making it the most frequent variable measured in older adults. On the other hand, its measurement is extremely important, since a sedentary lifestyle increases the risk of heart and metabolic diseases, which already have a high incidence rate in this population. In this way, the measurement of this variable is extremely important, since the diagnosis of movement and activity allows an early intervention in the sense of promoting health in older adults [19,23,30,31,46,47]. 

Biological signals related to the cardiac and respiratory systems, such as HR, RR, and oxygen saturation, also presented a high frequency of measurement indication in the age group under study. This factor is again due to the need to monitor the health status of older adults, assessing vital signals, which are essential for understanding the proper functioning of the cardiorespiratory system. In this population, the cardiovascular system and respiratory system are more fragile and probably experiencing pathological changes; better monitoring of older adults allows early diagnosis and intervention [21,26,27,28,31,48,49]. 

Other variables, which are not new, but appeared less often, such as sleep, glucose levels, and fall risk, are also extremely important for the population under study. All variables report the health status of older adults; so, their monitoring is also relevant and gives health professionals information about the health status of the older adult [22,28,29,50,51]. 

Different biological and environmental signals were measured through different types of wearable/sensors. Through this review, we saw that a wearable group included different types of sensors, making it possible to measure several signals with a single device. For example, a waist-worn device can be used to monitor the HR, ECG, RR, and PA, measuring the state of the cardiac, respiratory, and movement systems. 

In relation to body areas for the wearables, most of the studies pointed to the thigh, wrist, and waist as the most suitable places to measure biological signals. Evidently, the systems tend to be increasingly simple, user-friendly, and less intrusive; so the individual can carry out their normal tasks throughout the day, without the system interfering. In this sense, the places identified through different devices, allowed the user to quickly forget their use, enabling monitoring and evaluation in a real context without feeling the pressure of being evaluated, avoiding the modification of values [21,26,27,28,31,48,49,52].

Reliability and validity are considered two of the main measurement properties of instruments [53]. In this review, the steps variable was the only one with values presented for reliability and validity, and the values found agreed with Evenson et al. (2015) [54]. In relation to the other variables, such as posture, daily activity time, and sleep, in which it was possible to identify the reliability and validity values, these appeared to be acceptable [53,54,55]. The PA level and cardiac rhythm also seemed to have acceptable validity values. These are the oldest variables in terms of health investigation, which has meant more development time and thus better psychometric characteristics, due to their development and continuous improvement.

Only two reviews [24,34] assessed environmental signals. These reviews demonstrated a lack of information in scientific data about how environmental signals modify biological signals or the quality of life/health status of the users. The most common measures used were related to physiological monitoring, functional monitoring, emergency detection, and safety/security monitoring. These are very important measures to improve the quality of life/health status of the user, especially in cases where safety is very important to guarantee their health status [56].

Understand the methodological quality of the studies is important. In umbrella reviews, the quality of the original studies included in the reviews, as well as the quality of the reviews themselves, should be assessed. The risk of bias and the quality of the studies were not always defined, which means that there was no knowledge about the relevance/quality of the studies included in the reviews, which made interpretation of the results difficult; however, in relation to the reviews’ methodological quality, they were average to good quality. The quality of the studies was dispersed, ranging from very low to high quality, making comparisons difficult. In line with these limitations, some factors should be considered in the interpretation of the results of the present study. Although wearables are for everyone, some studies did not mention, did not characterize, or included different populations (different ages, different health conditions, and community-dwelling or institutionalized older adults). These factors can cause variations in the health-related biological signals. Moreover, different health conditions and different environments cause variations in the functioning and performance of the different wearables, making the comparation between studies very difficult [4,57,58,59,60,61,62,63,64]. Differences between the studies in terms of the aims and sample sizes (number of studies included in the original reviews) made it difficult to compare them. For example, checking the reliability and validity of different systems versus finding the usability of system and studies with a large sample (73) versus a small sample (7) indicated high heterogeneity between the studies and between their methodologies. Future studies comparing various uses of wearables, their advantages, and disadvantages in different age groups, living conditions, and specific pathologies are required. Moreover, more studies assessing the systems’ effectiveness for older adults’ health are required. 

## 5. Conclusions

The results of the present review demonstrated that the most frequent body regions used to assess older adults’ biological signals were the wrist, waist, and chest. The signals collected at these regions were mostly used to assess PA (through various variables such as energy expenditure, posture, and METs) and cardiovascular variables (through signals such as HR and cardiac rhythm). The environmental systems were used to assess environmental features; however, the health-related biological signals of older adults were also measured. This monitoring strategy had the advantage of monitoring the elderly person in the place/house, where the older adult was. Among all biological signals, the most frequent were ECG, temperature, HR, and body mass. These systems used a wide variety of sensors (mechanical, acoustic, optical, and air-related), and among the most frequent environment signals assessed, we highlight gas (density and saturation) and sound.

Despite providing a global overview of the monitoring of older adults’ biological signals, the divergence observed between the studies included in the present review limited the comparison between different systems. Therefore, future studies with more specific criteria regarding study methodology are required. Moreover, while the psychometric properties of some systems were presented, the study of these properties needs to be extended to the other systems. This information will help the decision-making process regarding the selection of the system to be used.

## Figures and Tables

**Table 1:** Characteristics of each review included in this umbrella review, with the authors and year published, the city and country in which the review was conducted, the aim, the number of included studies (n) and respective countries, and the databases searched.

Authors and Year Published	City, Country	Aim: To Review the:	Studies Included in the Review			Databases Searched
			n	Type	Continent/Countries	
Straiton et al. (2018) [30]	Sidney, Australia	Validity and reliability of consumer-grade activity trackers in community-dwelling older adults.	7	OBS	Europe, Australia, USA, Canada	MEDLINE, CINAHL, COCHRANE, Central Register of Controlled Clinical Trials
Lim et al. (2018) [17]	Wessex, UK	Measures of hospitalized older adults’ physical activity.	18	RCT, nRCT	USA, Norway, Australia, Denmark, France, Israel	MEDLINE, EMBASE, CINAHL, AMED
Feehan et al. (2018) [29]	Richmond, Canada	Accuracy of Fitbit activity trackers in controlled and free-living settings.	67	-	North America, Western Europe, South Asia, and Australia	PubMed, EMBASE, CINAHL, SPORTDiscus, Google Scholar
Khan et al. (2020) [33]	Ziauddin, Pakistan	PA monitors among the sedentary population.	16	RCT	-	PubMed, Google Scholar, Google, MEDLINE, Cochrane Library
Prasitlumkum et al. (2021) [26]	California, USA	Accuracy of an atrial fibrillation diagnosis by smart gadgets/wearable devices.	21	OBS	Norway, Netherlands, Finland, UK, USA, Hong Kong, Belgium, Germany, Italy	MEDLINE, EMBASE, Cochrane
Alharbi et al. (2019) [31]	Sydney, Australia	PA, ECG, and vital signals from wearable sensors among older adults.	20	-	Italy, USA, Canada, Australia, Germany, Denmark, Japan, Netherlands	CINAHL, MEDLINE, PubMed,
Dasenbrock et al. (2016) [18]	Oldenburg, Germany	Potential of IT and sensor technology to assess the functionality and mobility of pre-frail and frail older adults.	28	CSS *	-	PubMed, Cochrane Library
Kristoffersson and Lindén (2020) [19]	Västerås, Sweden	Use of wearable body sensors for health monitoring.	73	OBS, CSS, CC, CO	Africa, Australia, Austria, Belgium, Brazil, Canada, China, Colombia, Estonia, France, Germany, Greece, India, Ireland, Italy, Japan, Jordan, Korea, Macedonia, Portugal, Saudi Arabia, Slovenia, South Africa, Spain, Sweden, Switzerland, Taiwan, Netherlands, Tunisia, UK, United Arab Emirates, USA	Web of Science Core Collection, MEDLINE, Scopus, ScienceDirect, Academic Search Elite, ACM Digital Library, IEEE Xplore
Moore et al. (2021) [20]	Cork, Ireland	User experience and acceptance after a multi-day trial with a wearable device.	20	-	Western countries	CINAHL, APA PsycINFO, PubMed, EMBASE
Clark et al. (2019) [21]	Devon, UK	Accuracy of automated devices for measuring BP, with or without AF detection.	13	RCT, OBS	Pacific Northwest, Slovakia, Padua, Canada, England, Poland, Norway, Lithuania, Greece, Scotland, Western General, Spain	MEDLINE, EMBASE
Mattishent and Loke (2018) [22]	Norwich, UK	Use of CGM in older patients.	9	RCT, OBS	USA, Japan, Germany, Canada, Netherlands	SCI Web of Science, Ovid SP, MEDLINE, EMBASE
Vavasour et al. (2021) [23]	Dundalk, Ireland	Methods of using wearable sensors to assess frailty in older adults.	29	OBS	-	Medline, Science Direct, Scopus, CINAHL
Wang et al. (2021) [24]	Germany	Current sensor technology for unobtrusive in-home monitoring	55	OBS	-	ACM Digital Lib, IEEE Xplore, PubMed, Scopus
Olson and Lockhart (2021) [27]	Arizona, USA	Use of wearable sensors to predict fall risk.	54	PRO	-	PubMed
Heesch et al. (2018) [32]	Brisbane, Australia	Validity and reliability of accelerometers for the assessment of sedentary behavior in older adults.	15	RCT, OBS	USA, Switzerland, Canada, Australia, Norway, Germany, UK	EMBASE, PubMed, EBSCOhost
Bezold et al. (2021) [25]	Karlsruhe, Germany	Current research on wearable sensors for fall risk assessment in older adults with or without cognitive impairment.	28	CSS mixed design	-	PubMed, Scopus, Web of Science
Vegesn et al. (2017) [28]	Philadelphia, USA	Key trends associated with RPM via noninvasive digital technologies.	62	RCT, OBS, CS	France, USA, Italy, China, Australia, Spain, Denmark, Canada, Taiwan, Germany, Korea, Switzerland, Australia	EMBASE, Ovid, MEDLINE
Revathi et al. (2019) [34]	Chennai, India	Medical equipment widely used in the hospital.	39	-	-	Scopus journals and a survey report by WHO on telecommunication and information technology

* development of a monitoring system.

**Table 2:** Methodological Quality Assessment using AMSTAR 2.0.

Authors of the Review (Year)	1	2	3	4	5	6	7	8	9	10	11	12	13	14	15	16	Overall Quality
Straiton et al. (2018) [30]	Yes	Partial Yes	No	Yes	Yes	-	No	Yes	Yes	Yes	Not Applicable	Not Applicable	Yes	Yes	No	Yes	Moderate
Lim et al. (2018) [17]	Yes	Yes	No	Yes	Yes	Yes	No	Yes	Yes	Yes	Not Applicable	Not Applicable	Yes	Yes	Yes	Yes	Moderate
Feehan et al. (2018) [29]	Yes	Yes	Yes	Yes	Yes	Yes	No	Yes	Yes	Yes	Not Applicable	Not Applicable	Yes	Yes	Yes	Yes	High
Khan et al. (2020) [33]	No	Partial Yes	No	No	Yes	No	No	No	Yes	No	No	Not Applicable	No	Yes	Yes	No	Low
Prasitlumkum et al. (2021) [26]	Yes	Yes	No	Yes	Yes	No	Yes	Yes	Yes	Yes	Yes	Yes	Yes	Yes	Yes	Yes	Moderate
Alharbi et al. (2019) [31]	Yes	Yes	No	Yes	Yes	No	No	Yes	No	Yes	Not Applicable	Not Applicable	No	Yes	No	Yes	Low
Dasenbrock et al. (2016) [18]	Yes	Yes	No	Yes	Yes	No	Yes	Yes	No	No	Not Applicable	Not Applicable	No	Yes	No	Yes	Low
Kristoffersson and Lindén (2020) [19]	Yes	Yes	No	Yes	No	No	Yes	Yes	No	No	Not Applicable	Not Applicable	No	No	No	Yes	Low
Moore et al. (2021) [20]	Yes	Yes	Yes	Yes	Yes	Yes	Yes	No	No	Yes	Not Applicable	Not Applicable	No	Yes	No	Yes	Low
Clark et al. (2019) [21]	Yes	Yes	Yes	Yes	Yes	Yes	Yes	No	Yes	Yes	Yes	Yes	Yes	Yes	No	Yes	High
Mattishent and Loke (2018) [22]	Yes	Yes	Yes	Yes	Yes	Yes	Yes	No	Yes	Yes	Not Applicable	Not Applicable	Yes	Yes	Yes	Yes	High
Vavasour et al. (2021) [23]	Yes	Yes	Yes	Yes	No	No	Yes	Yes	Yes	Yes	Not Applicable	Not Applicable	No	Yes	No	Yes	Moderate
Wang et al. (2021) [24]	Yes	Yes	Yes	Yes	Yes	Yes	No	Yes	No	Yes	Not Applicable	Not Applicable	No	No	No	Yes	Low
Olson and Lockhart (2021) [27]	No	Yes	Yes	Yes	No	No	Yes	No	No	No	Not Applicable	Not Applicable	No	No	No	No	Low
Heesch et al. (2018) [32]	Yes	Yes	Yes	Yes	Yes	Yes	Yes	Yes	No	Yes	Not Applicable	Not Applicable	No	Yes	No	Yes	High
Bezold et al. (2021) [25]	Yes	Yes	Yes	Yes	Yes	Yes	Yes	Yes	No	Yes	Not Applicable	Not Applicable	No	No	No	Yes	Moderate
Vegesna et al. (2017) [28]	Yes	Yes	Yes	Yes	Yes	No	No	Yes	Yes	Yes	Not Applicable	Not Applicable	No	No	No	Yes	Low
Revathi et al. (2019) [34]	No	Yes	No	Yes	No	No	No	No	No	Yes	Not Applicable	Not Applicable	No	No	No	Yes	Critically Low

**Table 3:** Characteristics of the studies’ participants.

Authors of the Review (Year)	n	Number of Older Adults	Age (Years) (Mean ± Standard Deviation or Range)	Health Condition (Healthy/Pathology)	Community-Dwelling/Institutionalized Older Adults
Straiton et al. (2018) [30]	7	290	70.2 ± 4.8	CHD, COPD, Absence of Specific Disease-Based Criteria	F, C, H
Lim et al. (2018) [17]	18	-	-	Neurologic Diseases	F, C, H
Feehan et al. (2018) [29]	67	2441	21–84	Healthy, Chronic Diseases, Mobility Limitations	F, C
Khan et al. (2020) [33]	16	2542	>18	Sedentary	F
Prasitlumkum et al. (2021) [26]	21	17,131	73.7 ± 9.1	Healthy, Cardiovascular Diseases, Metabolic Diseases	F, C, H
Alharbi et al. (2019) [31]	20	3741	69 ± not revealed	High Risk of Cardiovascular Disease, Chronic Obstructive Pulmonary Disease, Cardiac Patients, Postoperative Surgical Patients	F, C
Dasenbrock et al. (2016) [18]	28	1917	21–90	Frail, Pre-Frail, or Robust	F, H
Kristoffersson and Lindén (2020) [19]	73	1628	>16	Healthy, Respiratory Diseases, Cardiovascular Diseases, Metabolic Diseases, Neurological Diseases	F, C
Moore et al. (2021) [20]	20	349	51–94	Healthy, Previous Breast Cancer, Obesity, Resolving Heart Failure, Parkinson’s Disease, Alzheimer and Dementia, Walking Aids	F, H
Clark et al. (2019) [21]	13	-	68–83	Atrial Fibrillation, Hypertension and Normotension	F, C, H
Mattishent and Loke (2018) [22]	9	989	70	Diabetes	F
Vavasour et al. (2021) [23]	29	7491	18–90	-	F, C, H
Wang et al. (2021) [24]	55	>843	>20	Heart Disease, Healthy, Hearing Impairment, Walking, Abnormalities, Alzheimer’s Disease, Mild Cognitive, Impairment, Cognitive Problem/Difficulties, Parkinson’s Disease, Risk of Cognitive Difficulties, Type II Diabetes, Stroke Survivors	F
Olson and Lockhart (2021) [27]	54	5–300	-	Non-Frail/Non-Fallers, Parkinson’s Disease Fallers, Dementia Fallers, Stroke Fallers, Diabetes Fallers, Cardiac Patients Frail	F, C
Heesch et al. (2018) [32]	15	>11,173	61–78	Healthy	F, C
Bezold et al. (2021) [25]	28	2896	68–86 Control: 21–35	Dementia, Fallers and Non-Fallers	F, H
Vegesna et al. (2017) [28]	62	8348	Over 20	Respiratory Diseases, Weight Management, Metabolic Diseases, Cardiovascular Diseases, Cancer, Neurological, Psychological, Sleep Disorders, Substance Abuse	F
Revathi et al. (2019) [34]	39	-	-	-	-

**Table 4:** Biological Signals.

Category	Biological Signal	Health Status Information of the Biological Signal (Clinical Meaning)	Cutoffs	Device Placement	Author (Year)
Movement related variables	METs by steps	Level of PA and SB of participants	PA: Light = 1.1–2.9 METs Moderate = 3.0–5.9 METs Vigorous ≥ 6.0 METs	Ankle	Lim (2018) [17] Straiton (2018) [30] Feehan (2018) [29] Alharbi (2019) [31] Moore (2021) [20]
				Waist	Straiton (2018) [30] Feehan (2018) [29] Alharbi (2019) [31] Moore (2021) [20]
				Wrist	Straiton (2018) [30] Feehan (2018) [29] Alharbi (2019) [31] Vavasour (2021) [23]
				Thigh	Lim (2018) [17] Feehan (2018) [29] Alharbi (2019) [31]
				Sternum	Feehan (2018) [29] Vavasour (2021) [23]
				Chest	
				Torso	
				Bra	
				Upper arm	
				Lumbar Spine	Vavasour (2021) [23]
				Not reported by the authors	Kristoffersson (2020) [19] Olson (2021) [27] Revathi (2019) [34]
Movement variables	METs by cpm	Level of PA and/or SB of participants	SB: <1.5 METs, <100 cpm PA: Light: 1.5–3.0 METs, 100–1040 cpm Moderate: ≥3.0 METs, 1041–1951 cpm Vigorous: >1052 cpm	Waist	Lim (2018) [17], Alharbi (2019) [31], Dasenbrock (2016) [18], Vavasour (2021) [23]
				Wrist	Khan (2020) [33] Alharbi (2019) [31] Moore (2021) [20]
				Foot	Dasenbrock (2016) [18] Khan (2020) [33]
				Hip	Khan (2020) [33] Vavasour (2021) [23]
				Thigh	Khan (2020) [33]
				Lower Back	
				Chest	
				-	Kristoffersson (2020) [19]
	Posture	Body organization	-	Thigh	Lim (2018) [17]
				Chest	
	Energy expenditure (kcal/kg/day)	Level of PA and/or SB of participants	Light PA: <6.2 kcal/kg/day for men <7.13 kcal/kg/day for women	Hip	Feehan (2018) [29] Vavasour (2021) [23]
				Waist	Feehan (2018) [29] Bezold (2021) [25]
				Wrist	Feehan (2018) [29] Alharbi (2019) [31] Bezold (2021) [25]
					Straiton (2018) [30]
				Torso	Feehan (2018) [29]
				Lower Back	Bezold (2021) [25]
				Upper Legs, Chest, Foot	
Movement Variables	METs by cpm ECG intervals	Level of PA and/or SB of participants Fall risk Frailty	SB: VA < 100 cpm VM < 200 cpm. Sedentary activities 1-s (<1 to <10 in increments of 1 count/s) 15-s (<1 to <100 in increments of 5 counts/15 s) 60-s (<1 to <400 in increments of 25 cpm) Sedentary time: <1.5 METs <100 cpm <270 kcal/week for women <383 kcal/week for men PA Light: 1.5–3.0 METs, 100–1040 cpm Moderate: ≥3.0 METs, 1041–1951 cpm Vigorous: >1052 cpm A cutoff value of 1.58 m/s gait speed discriminates between HIGH RISK OF FALL and LOW RISK OF FALL. Fallers had lower average R-R intervals (time between R waves of the ECG), lower variability in R-R duration, and increased power in the low frequency component of the heart wave during continuous monitoring. “Frail: longer transition duration, decreased smoothness of transition pattern and dynamic of trunk movement Frail: acceleration and balance parameters in the 10 m extended timed get up and go test”	-	Vavasour (2021) [23] Olson (2021) [27] Bezold (2021) [25] Dasenbrock (2016) [18] Heesch (2018) [32]
Cardiovascular Variables	Cardiac Rhythm	Early detection of AF	Incidence of newly diagnosed AF defined as ≥30 s of AF or flutter detected by tracker. Each AF episode defined as presence of ≥30 s of continuous AF during monitoring.	Fingertip	Prasitlumkum (2021) [26]
				Wrist	Prasitlumkum (2021) [26]
				Chest	Prasitlumkum (2021) [26] Alharbi (2019) [31]
				Facial	Prasitlumkum (2021) [26]
				Fingertip	Prasitlumkum (2021) [26]
				Arm	Clark (2019) [21]
	HR/Pulse/Heart Rate Variability	-	Bradycardia (HR < 50 bpm) Tachycardia (HR > 100 bpm)	Wrist	Alharbi (2019) [31]
				Chest/ Thorax	Alharbi (2019) [31] Vegesna (2017) [28]
				Arm	Vegesna (2017) [28]
				-	Olson (2021) [27]
					Revathi (2019) [34]
					Kristoffersson (2020) [19]
	ECG	-	-	Chest	Alharbi (2019) [31]
				-	Kristoffersson (2020) [19]
	RR	-	Bradypnea (RR < 12 bpm) Tachypnea (RR > 20 bpm)	Chest	Alharbi (2019) [31]
				-	Kristoffersson (2020) [19]
				Chest	Vegesna (2017) [28]
	BP	-	-	-	Kristoffersson (2020) [19]
				Arm	Clark (2019) [21]
				-	Revathi (2019) [34]
Other biological signal variables	EMG	-	-	Waist, Arm, and Leg	Dasenbrock (2016) [18]
	GPS	-	-	Waist	Dasenbrock (2016) [18]
				Foot	
	BT	-	-	-	Kristoffersson (2020) [19]
				Arm	Vegesna (2017) [28]
				Chest	
				-	Revathi (2019) [34]
	SpO_2_	-	-	-	Kristoffersson (2020) [19]
				Arm	Vegesna (2017) [28]
				Chest	
				-	Revathi (2019) [34]
	Accelerometry	Fall risk detection	A faller was defined as a person having at least one fall over a certain period of time, usually the past or prospective 12 months.	Torso	Moore (2021) [20]
				-	Olson (2021) [27]
		Sleep	-	Wrist	Feehan (2018) [29]
				-	Kristoffersson (2020) [19]
				-	Revathi (2019) [34]
		Stress	-	-	Kristoffersson (2020) [19]
	Glucose levels	-	-		Mattishent (2018) [22]
				Arm	Vegesna (2017) [28]
				Chest	
					Revathi (2019) [34]
	Weight	-	-	-	Vegesna (2017) [28]

**Table 5:** Sensor Type.

Authors (year)	Sensor Type
Straiton et al. (2018) [30]	Consumer-grade activity trackers
Lim et al. (2018) [17]	Accelerometer
Feehan et al. (2018) [29]	Accelerometer
Khan et al. (2020) [33]	Accelerometer
Prasitlumkum et al. (2021) [26]	ECG sensor
Alharbi et al. (2019) [31]	Accelerometer, consumer-grade activity tracker, pedometer
Dasenbrock et al. (2016) [18]	Cameras, force platforms and foot switch, triaxial accelerometers, gyroscope, pressure sensors, pedometers, grip ball, motion sensors, bed sensors, stove sensors
Kristoffersson and Lindén (2020) [19]	Accelerometer, electrocardiography sensor, pressure sensor
Moore et al. (2021) [20]	Accelerometer, pedometer, motion sensor
Clark et al. (2019) [21]	Sphygmomanometer, oximeter
Mattishent and Loke (2018) [22]	Continuous glucose monitor
Vavasour et al. (2021) [23]	IMUs
Wang et al. (2021) [24]	Accelerometer, pressure sensor, contact sensor, ECG sensor, gas/dust sensor, camera, ultrasonic sensor, water flow sensor
Olson and Lockhart (2021) [27]	IMUs, barometer, pressure insoles, ECG sensor, respiratory monitor
Heesch et al. (2018) [32]	Accelerometer, temperature sensor, ambient light sensor
Bezold et al. (2021) [25]	Sensor-based balance, IMUs
Vegesna et al. (2017) [28]	Spirometry, optical sensor, ECG sensor, oximeter, sphygmomanometer and FEV1 monitors, IMUs, pedometer
Revathi et al. (2019) [34]	IMUs, optical, photoConductive, piezo-electric based, pressure, radar, radiofrequency, sonar, surface, electromyography, thermistor, thermoelectric effects, ultrasonic, photoplethysmography

**Table 6:** Psychometric properties of the health-related biological signals—device—sensor—device placement.

Biological Signal	Local	Psychometric Properties		Sensor Type	Author (Year)
		Validity	Reliability		
Steps	Ankle	r = 0.76	ICC = 0.99	Accelerometer	Lim (2018) [17]
		-	Percentage error < 10% at 0.4–0.9 m/s	Consumer-grade activity trackers	Straiton (2018) [30]
	Waist	r = 0.90	ICC = 0.60–0.96	Consumer-grade activity trackers Triaxial accelerometers	Straiton (2018) [30] Dasenbrok (2016) [18]
			Percentage error < 10% at 0.8–0.9 m/s		
	Wrist	r = 0.96	ICC = 0.15	Consumer-grade activity trackers	Straiton (2018) [30]
	Thigh	-	Limits of agreement = −2.01 to 16.54 Absolute percent error = 40.31	Accelerometer	Lim (2018) [17]
	Torso		Percentage error < −10.6%	Accelerometer	Feehan (2018) [29]
Daily activity time	Wrist	r = 0.25	Percentage error < −8.6%	Consumer-grade activity trackers	Straiton (2018) [30] Feehan (2018) [29]
	Ankle	-	Percentage error < 2.9%	Accelerometer	Feehan (2018) [29]
PA level	Waist	r = 0.780	Percentage error = 10%	Accelerometer	Lim (2018) [17]
	Wrist	r = 0.965	-	Accelerometer	Khan (2020) [33]
	Foot	r = 0.955			
	Hip	r = 0.978			
	Thigh	r = 0.971			
	Lower Back	r = 0.968			
	Chest	r = 0.969			
Posture	Thigh	-	Limits of agreement = −2.01 to 16.54 Absolute percent error = 40.31	Accelerometer	Lim (2018) [17]
Sleep	Wrist	-	Percentage error < −8.6%	Accelerometer	Feehan (2018) [29]
Energy expenditure	Wrist	-	Percentage error < −8.6%	Accelerometer	Feehan (2018) [29]
		r = 0.74	-	Consumer-grade activity trackers	Straiton (2018) [30]
	Torso	-	Percentage error < −10.6%	Accelerometer	Feehan (2018) [29]
Cardiac Rhythm	Fingertip	-	Accuracy—94.0–97.4 Sensitivity—87.0–100.0 Specificity—84.9–98.8	ECG sensor	Prasitlumkum (2021) [26]
	Wrist	-	Accuracy—89.2–99.2 Sensitivity—75.0–93.7 Specificity—84.0–98.2		
	Chest	-	Accuracy—95.7 Sensitivity—95.3 Specificity—96.0		
	Facial	-	Accuracy—95.4 Sensitivity—94.7 Specificity—95.8		
	Fingertip	-	Accuracy—92.0–96.1 Sensitivity—93.1–95.6 Specificity—90.9–96.6		
Movement (free-living activities	Waist	-	ICC: 0.80–5 days ICC: 0.95–21 days	Accelerometer	Heesch (2018) [32]
	Hip	-	ICC: 0.74 (0.65, 0.80) Sensitivity: 61–92% Specificity: 43–91%		
	Wrist	-	Sensitivity: 78–82% Specificity: 70–78%		
	Thigh	-	Sensitivity: 99.3–99.9% Specificity: 99.2–99.7%		

**Table 7:** Environmental Signals, retrieved from: Wang et al. 2021 [24,34].

Functions					
Phy Fx Em SaSe					
Data					
Physiology Body temperature, BP, Body mass, ECG, HR, RR		Behavior Activity level, Computer usage, Gait parameters, Phone usage, Presence, Time spent on activities, Out of home, Walking speed		Environment Gas concentration, Humidity, Temperature, Sound	
Locations					
Electrical appliances Coffee machine, Computer, Fridge, Stove/oven, Lamp, Microwave oven, Television (TV), Phone, Radio, Water kettle		Static facilities Floor (specific area), Wall (specific), Window, Sink, Toilet, Chair/sofa/couch, Bed, Door, Shelf/cabinet/drawer		Rooms Living room, Kitchen, Bedroom, Bathroom, Hallway, Study room	
Unobtrusive Sensors					
Acoustic Microphone Ultrasonic sensor	Air-related Gas/dust sensor Humidity sensor Thermometer	Mechanical Accelerometer Bed sensor Scale Pressure sensor Vibration sensor	Electromagnetic Contact sensor Electrocardiography sensor Power meter Radar	Optical PIR motion sensor Infrared camera Video camera Depth camera	Unclassified Water flow sensor Computer monitoring (software) Phone monitor

## Data Availability

Not applicable.

## Abbreviations

BT	body temperature
HR	heart rate
RR	respiratory rate
BP	blood pressure
StO_2_	pulse oxygenation
BG	blood glucose
PRISMA	Preferred Reporting Items for Systematic Reviews and Meta-Analyses
JF and JM	reviewers
ASP	reviewer consulted in case of doubt
AMSTAR	Assessing the Methodological Quality of Systematic Reviews 2.0
OBS	observational
USA	United States of America
UK	United Kingdom
RCT	randomized control trial
nRCT	non-randomized control trial
CSS	cross-sectional
CC	case control
CO	cross over
PRO	prospective
CS	case series
*	development of a monitoring system
n	number of studies
-	not reported
PA	physical activity
ECG	echocardiography
IT	information technology
AF	atrial fibrillation
CGM	continuous glucose monitoring
RPM	remote patient monitoring
F	free-living
C	controlled
H	hospitalized
CHD	coronary heart disease
COPD	chronic obstructive pulmonary disease
SD	standard deviation
SB	sedentary behavior
METs	metabolic equivalent of task
cpm	counts per minute
VA	vertical axis
VM	vertical magnitude
bpm	beats per minute
GPS	global positioning system
IMUs	inertial motion units
FEV1	first second of forced expiration
ICC	intraclass coefficient correlation
r	coefficient correlation
Phy	physiological monitoring
Fx	functional monitoring
Em	emergency detection
SaSe	safety/security monitoring
TV	television

## Appendix A. Search String by Database

**Pubmed** OLDER ADULTS (“aged”[MeSH Terms] OR “aged”[Title/Abstract] OR “elder*”[Title/Abstract] OR “aged, 80 and over”[MeSH Terms] OR “older adult*”[Title/Abstract] OR “older person*”[Title/Abstract] OR “centenarian*”[Title/Abstract] OR “nonagenarian*”[Title/Abstract] OR “octogenarian*”[Title/Abstract]) BIOLOGICAL AND ENVIRONMENTAL SIGNALS (“Vital Signals”[MeSH Terms] OR vital[Title/Abstract] OR “vital sign*”[Title/Abstract] OR “vital function*”[Title/Abstract] OR “vital parameter*”[Title/Abstract] OR “biological sign*” [Title/Abstract] OR “physical activity”[Title/Abstract] OR “sedentary behavior”[MeSH Terms] OR “sedentary behavior”[Title/Abstract] OR “cardiorespiratory fitness”[MeSH Terms] OR “cardiorespiratory fitness”[Title/Abstract] OR “electrocardiography”[MeSH Terms] OR “electrocardiography”[Title/Abstract] OR “blood glucose”[MeSH Terms] OR “blood glucose”[Title/Abstract] OR “galvanic skin response”[MeSH Terms] OR “galvanic skin response”[Title/Abstract] OR “oximetry”[MeSH Terms] OR “oximetry”[Title/Abstract] OR “Humidity”[MeSH Terms] OR “humidity”[Title/Abstract] OR “Temperature”[MeSH Terms] OR “temperature”[Title/Abstract] OR “lighting”[MeSH Terms] OR “lighting”[Title/Abstract]) TELEMONITORING (“wearable electronic devices”[MeSH Terms] OR “wearable electronic devices” [Title/Abstract] OR “wearable devices”[Title/Abstract] OR “wearable technology”[Title/Abstract] OR “sensor”[Title/Abstract] OR “device*”[Title/Abstract] OR “wearable”[Title/Abstract] OR Internet of Things[MeSH Terms] OR “Internet of Things”[Title/Abstract] OR “Remote continuous monitoring”[Title/Abstract] OR “wireless device”[Title/Abstract] OR “patch”[Title/Abstract] OR “appliance”[Title/Abstract] OR “portable”[Title/Abstract] OR “Monitoring, Physiologic”[MeSH Terms] OR “Monitoring, Physiologic”[Title/Abstract] OR “tracker*”[Title/Abstract] OR “Environmental Monitoring”[MeSH Terms] OR “Environmental Monitoring” [Title/Abstract] OR “Environmental Quality” [Title/Abstract]) STUDY DESIGN “Review”[Publication Type] OR “Review” [Title/Abstract] OR “review literature as topic”[MeSH Terms] OR “Systematic review”[Publication Type] OR “Systematic reviews as topic”[MeSH Terms] OR “systematic review”[Title/Abstract] OR “Meta-Analysis”[Publication Type] OR “Meta analysis as Topic”[MeSH Terms] OR “Meta analysis”[Title/Abstract] OR “Meta-analysis as Topic”[MeSH Terms]
**COCHRANE DATABASE OF SYSTEMATIC REVIEWS** Search Hits #1MeSH descriptor: [Aged] explode all trees 215061 #2”elder*”:ti,ab,kw 60362 #3MeSH descriptor: [Aged, 80 and over] explode all trees 54685 #4”older adult*”:ti,ab,kw 983 #5”older person*”:ti,ab,kw 142 #6”centenarian*”:ti,ab,kw 6 #7”nonagenarian*”:ti,ab,kw 5 #8”octogenarian*”:ti,ab,kw 31 #9#1 OR #2 OR #3 OR #4 OR #5 OR #6 OR #7 OR #8 259899 #10MeSH descriptor: [Vital Signals] explode all trees 37510 #11”vital”:ti,ab,kw 28486 #12”vital sign*”:ti,ab,kw 4493 #13”vital function*”:ti,ab,kw 9 #14”vital parameter*”:ti,ab,kw 11 #15”biological sign*”:ti,ab,kw 1 #16”physical activity”:ti,ab,kw 34481 #17”sedentary behavior”:ti,ab,kw 2372 #18”cardiorespiratory fitness”:ti,ab,kw 2297 #19MeSH descriptor: [Sedentary Behavior] explode all trees 1240 #20MeSH descriptor: [Cardiorespiratory Fitness] explode all trees 345 #21MeSH descriptor: [Electrocardiography] explode all trees 8928 #22”Electrocardiography”:ti,ab,kw 12256 #23MeSH descriptor: [Blood Glucose] explode all trees 16796 #24”blood glucose”:ti,ab,kw 32059 #25”galvanic skin response”:ti,ab,kw 782 #26MeSH descriptor: [Galvanic Skin Response] explode all trees 659 #27MeSH descriptor: [Oximetry] explode all trees 1049 #28”oximetry”:ti,ab,kw 4106 #29MeSH descriptor: [Humidity] explode all trees 549 #30humidity:ti,ab,kw 1753 #31MeSH descriptor: [Temperature] explode all trees 4530 #32”temperature”:ti,ab,kw 22891 #33MeSH descriptor: [Lighting] explode all trees 240 #34”lighting”:ti,ab,kw 820 #35#10 OR #11 OR #12 OR #13 OR #14 OR #15 OR #16 OR #17 OR #18 OR #19 #20 OR #21 OR #22 OR #23 OR #24 OR #25 OR #26 OR #27 OR #28 OR #29 OR #30 OR #31 OR #32 OR #33 OR #34 158303 #36MeSH descriptor: [Wearable Electronic Devices] explode all trees 521 #37”wearable electronic devices”:ti,ab,kw 109 #38”wearable technology”:ti,ab,kw 102 #39”sensor”:ti,ab,kw 3640 #40”device*”:ti,ab,kw 47770 #41”wearable”:ti,ab,kw 1430 #42MeSH descriptor: [Internet of Things] explode all trees 1 #43”Internet of Things”:ti,ab,kw 46 #44”Remote continuous monitoring”:ti,ab,kw 3 #45”wireless device”:ti,ab,kw 27 #46”patch”:ti,ab,kw 6815 #47”appliance”:ti,ab,kw 2083 #48”portable”:ti,ab,kw 3418 #49”sensor”:ti,ab,kw 3640 #50MeSH descriptor: [Monitoring, Physiologic] explode all trees 12705 #51”Monitoring, Physiologic”:ti,ab,kw 2321 #52”tracker*”:ti,ab,kw 872 #53MeSH descriptor: [Environmental Monitoring] explode all trees 280 #54”Environmental Monitoring”:ti,ab,kw 230 #55Environmental Quality:ti,ab,kw 2862 #56#36 OR #37 OR #38 OR #39 OR #40 OR #41 OR #42 OR #43 OR #44 OR #45 OR #46 OR #47 OR #48 OR #49 OR #50 OR #51 OR #52 OR #53 OR #54 OR #55 76944 #57 #9 AND #35 AND #56 with Cochrane Library publication date from Jan 2016 to present, in Cochrane Reviews 19
**WEB OF SCIENCE** TS=Topic Searches for topic terms in the following fields within a record. Title Abstract Author Keywords Keywords Plus^®^ OLDER ADULTS TS=(“aged” OR “elder*” OR “older adult*” OR “older person*” OR **“centenarian*” OR “nonagenarian*” OR “octogenarian*”**) BIOLOGICAL AND ENVIRONMENTAL SIGNALS TS=(“vital” OR “vital sign*” OR “vital function*” OR “vital parameter*” OR “biological sign*” OR “physical activity” OR “sedentary behavior” OR “cardiorespiratory fitness” OR “electrocardiography” OR “blood glucose” OR “galvanic skin response” OR “oximetry” OR “humidity” OR “temperature” OR “lighting”) TELEMONITORING TS=(“wearable electronic devices” OR “wearable devices” OR “wearable technology” OR “sensor” OR “device*” OR “wearable” OR “Internet of Things” OR “Remote continuous monitoring” OR “wireless device” OR “patch” OR “appliance” OR “portable” OR “sensor” OR “Monitoring, Physiologic” OR “tracker*” OR “Environmental Monitoring” OR “Environmental Quality”) STUDY DESIGN TS=(“Review” OR “systematic review”[Title/Abstract] OR “Meta analysis” String Final (((TS=((“aged” OR “elder*” OR “older adult*” OR “older person*” OR “centenarian*” OR “nonagenarian*” OR “octogenarian*”))) AND TS=((“vital” OR “vital sign*” OR “vital function*” OR “vital parameter*” OR “biological sign*” OR “physical activity” OR “sedentary behavior” OR “cardiorespiratory fitness” OR “electrocardiography” OR “blood glucose” OR “galvanic skin response” OR “oximetry” OR “humidity” OR “temperature” OR “lighting”))) AND TS=((“wearable electronic devices” OR “wearable devices” OR “wearable technology” OR “sensor” OR “device*” OR “wearable” OR “Internet of Things” OR “Remote continuous monitoring” OR “wireless device” OR “patch” OR “appliance” OR “portable” OR “sensor” OR “Monitoring, Physiologic” OR “tracker*” OR “Environmental Monitoring” OR “Environmental Quality”))) AND TS=((“Review” OR “systematic review”[Title/Abstract] OR “Meta analysis”))
**JBI DATABASE OF SYSTEMATIC REVIEWS AND IMPLEMENTATION REPORTS** Population aged.sh. or aged.ab. or elder*.ab. or (aged, 80 and over).sh. or older adult*.ab. or older person*.ab. or centenarian*.ab. or nonagenarian*.ab. or octogenerian*.ab. or aged.ti. or elder*.ti. or older adult*.ti. or older person*.ti. or centenarian*.ti. or nonagenerian*.ti. or octagenerian*.ti. Signals “Vital signals”.sh. or Vital.ti. or Vital.ab. or “Vital sign*”.ti. or “Vital sign*”.ab. or Vital function*.ti. or Vital funciton*.ab. or Vital parameter*.ti. or Vital parameter*.ti. or Biological sign*.ti. or Biological sign*.ab. or Physical activity.ti. or Physical activity.ab. or Sedentary Behavior.sh. or Sedentary Behavior.ti. or Cardiorespiratory fitness.sh. or Cardiorespiratory fitness.ti. or blood glucose.sh. or blood glucose.ti. or galvanic skin response.sh. or galvanic skin response.ti. or oximetry.sh. or humidity.sh. or temperature.sh. or temperature.ti. or lighting.sh. or lighting.ti. or Sedentary Behavior.ab. or eletrocardiography.sh. or eletrocardiography.ti. or eletrocardiography.ab. or oximetry.ti. or oximetry.ab. or Cardiorespiratory fitness.ab. or blood glucose.ab. or galvanic skin response.ab. or humidity.ti. or humidity.ab. or temperature.ab. or lighting.ab Telemonitoring wearable electronic devices.sh. or wearable electronic devices.ti. or wearable electronic devices.ab. or wearable devices.ti. or wearable devices.ab. or wearable technology.ti. or wearable technology.ab. or sensor.ti. or sensor.ab. or device*.ti. or device*.ab. or wearable.ti. or wearable.ab. or internet of things.sh. or internet of things.ti. or internet of things.ab. or remote continuous monitoring.ti. or remote continuous monitoring.ab. or wireless device.ti. or wireless device.ab. or patch.ti. or patch.ab. or appliance.ti. or appliance.ab. or portable.ti. or portable.ab. or Monitoring, physiologic.sh. or Monitoring, physiologic.ab. or Monitoring, physiologic.ti. or tracker*.ti. or tracker*.ab. or environmental monitoring.sh. or environmental monitoring.ab. or environmental monitoring.ab. or environmental quality.ab. or environmental quality.ti. Publication type review.pt. or review.ti. or review.ab. or review literature as topic.sh. or systematic review.pt. or systematic reviews as topic.sh. or systematic review.ab. or systematic review.ti. or meta-analysis.pt. or meta-analysis as topic.ab. or meta-analysis as topic.sh. or meta-analysis as topic.ti (aged.sh. or aged.ab. or elder*.ab. or (aged, 80 and over).sh. or older adult*.ab. or older person*.ab. or centenarian*.ab. or nonagenarian*.ab. or octogenerian*.ab. or aged.ti. or elder*.ti. or older adult*.ti. or older person*.ti. or centenarian*.ti. or nonagenerian*.ti. or octagenerian*.ti.) AND (“Vital signals”.sh. or Vital.ti. or Vital.ab. or “Vital sign*”.ti. or “Vital sign*”.ab. or Vital function*.ti. or Vital funciton*.ab. or Vital parameter*.ti. or Vital parameter*.ti. or Biological sign*.ti. or Biological sign*.ab. or Physical activity.ti. or Physical activity.ab. or Sedentary Behavior.sh. or Sedentary Behavior.ti. or Cardiorespiratory fitness.sh. or Cardiorespiratory fitness.ti. or blood glucose.sh. or blood glucose.ti. or galvanic skin response.sh. or galvanic skin response.ti. or oximetry.sh. or humidity.sh. or temperature.sh. or temperature.ti. or lighting.sh. or lighting.ti. or Sedentary Behavior.ab. or eletrocardiography.sh. or eletrocardiography.ti. or eletrocardiography.ab. or oximetry.ti. or oximetry.ab. or Cardiorespiratory fitness.ab. or blood glucose.ab. or galvanic skin response.ab. or humidity.ti. or humidity.ab. or temperature.ab. or lighting.ab.) AND (wearable electronic devices.sh. or wearable electronic devices.ti. or wearable electronic devices.ab. or wearable devices.ti. or wearable devices.ab. or wearable technology.ti. or wearable technology.ab. or sensor.ti. or sensor.ab. or device*.ti. or device*.ab. or wearable.ti. or wearable.ab. or internet of things.sh. or internet of things.ti. or internet of things.ab. or remote continuous monitoring.ti. or remote continuous monitoring.ab. or wireless device.ti. or wireless device.ab. or patch.ti. or patch.ab. or appliance.ti. or appliance.ab. or portable.ti. or portable.ab. or Monitoring, physiologic.sh. or Monitoring, physiologic.ab. or Monitoring, physiologic.ti. or tracker*.ti. or tracker*.ab. or environmental monitoring.sh. or environmental monitoring.ab. or environmental monitoring.ab. or environmental quality.ab. or environmental quality.ti.
